# Cryogenic 3D printing of heterogeneous scaffolds with capability to spatially tune cellular morphology of mesenchymal stem cells for integrated osteochondral regeneration

**DOI:** 10.1016/j.mtbio.2025.102560

**Published:** 2025-11-15

**Authors:** Lu Bai, Chongzhou Fang, Yulong Qi, Chong Wang, Min Wang

**Affiliations:** a Department of Sport Medicine, Peking University Shenzhen Hospital, Shenzhen, Guangdong, 518036, China; b Central Laboratory, Peking University Shenzhen Hospital, Shenzhen, Guangdong, 518036, China; c Department of Medical Image, Peking University Shenzhen Hospital, Shenzhen, Guangdong, 518036, China; d School of Mechanical Engineering, Dongguan University of Technology, No.1 University Rd, Songshan Lake, Dongguan, Guangdong, 523808, China; e Department of Mechanical Engineering, The University of Hong Kong, Pokfulam Road, Hong Kong, SAR, 00852, China; f Institute of Science & Technology Innovation, Dongguan University of Technology, Songshan Lake, Dongguan, Guangdong, 523808, China

**Keywords:** Cryogenic 3D printing, Osteochondral scaffold, TGF-β1, MSC aggregate, Chondrogenic differentiation, Supramolecular hydrogel

## Abstract

The aging of population and frequent sport injury cause severe osteochondral tissue regression and injury. In recent years, various osteochondral tissue engineering scaffolds with a biomimetic structure have been fabricated to simultaneously induce chondrogenic and osteogenic differentiation of mesenchymal stem cells (MSCs) into targeting cells (i.e., hyaline chondrocytes and osteoblasts) at separate zones. However, the regenerated cartilage tissue is still inclined to exhibit a fibrocartilage state. To obtain correct cell phenotype at corresponding zone and hence improve the integrated osteochondral regeneration, in this study, a bi-phasic scaffold consisting of closely bonded osteogenic peptide/β-tricalcium phosphate/poly(lactic-co-glycolic acid) subchondral frame and TGF-β1 loaded shape memory polyester cartilage frame which was further dispensed with gelatin-based double-network dynamically crosslinked supramolecular hydrogel was fabricated via multi-material sequential cryogenic 3D printing. The osteochondral scaffolds were mechanically similar to native osteochondral tissue. The subchondral zone facilitated adhesion, expansion and osteogenic differentiation of MSCs *in vitro* while the supramoecular hydrogel in the cartilage zone allowed spontaneous MSC aggregation which was favorable for efficient chondrogenic differentiation. However, long-term delivery of TGF-β1 in the cartilage zone was still required to continuously maintain the aggregation state of cell aggregates and enhance the MSC chondrogenic differentiation into hyaline chondrocyte-like cells. The *in vivo* animal study showed that the implanted bi-phasic scaffolds can recruit endogenous bone marrow derived-MSCs and achieve integrated osteochondral regeneration with correct cell phenotypes in 3 month, demonstrating that supramolecular hydrogels with dynamic bonds, together with chondroinductive biochemical cues, can function as essential constituents of the cartilage compartment in 3D printed osteochondral scaffolds, enabling precise regulation and stabilization of cellular organizational architecture, thereby supporting coordinated and structurally integrated osteochondral regeneration.

## Graphical abstract

**Figure:**
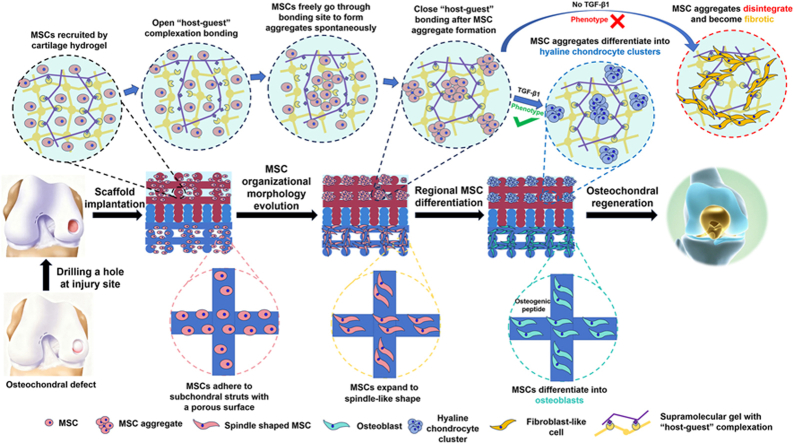

## 1 Introduction

Due to the increased aging population and the frequent occurrence of sports injury and accident trauma, more and more people suffer from degenerative osteoarthritis and traumatic osteochondral damage, which seriously restricts their motor functions and lowers their life quality [1,2]. It is known that native osteochondral tissue has a three-layer structure, where the hyaline cartilage layer, calcified interfacial layer and subchondral layer are closely bonded together [3]. In the hyaline cartilage layer, hyaline chondrocytes with a round shape are embedded in elastic hydrogel-like extracellular matrix (ECM) containing collagen II, hyaluronic acid, chondroitin sulfate, etc [4]. Differently, expanded osteoblasts are located in mechanically strong and structurally porous subchondral layer containing calcium phosphates and collagen I [5]. Conventional clinical methods for treating osteochondral injury mainly include arthroscopic debridement, microfracture bone marrow stimulation, autologous cartilage transplantation, and allogeneic cartilage transplantation [6,7]. These methods can to some degree mitigate inflammation, deliver endogenous mesenchymal stem cells (MSCs) to the lesion site or regenerate osteochondral tissue, however, their wide applications are restricted by their drawbacks such as easy loss of endogenous MSCs, limited osteochondral tissue donor sources, immune rejection and infectious diseases. Therefore, developing more advanced strategies to treat osteochondral defects is of great significance.

In recent years, tissue engineering scaffolds have been deemed as promising biomaterials to simulate the ECM of cells in native tissues to activate and regulate endogenous regeneration, providing effective means to assist and promote tissue repair [8,9]. To date, three-dimensional (3D) printing has been considered one of the most potent manufacturing techniques to produce tissue engineering scaffolds with customized shape, tailored microstructure and controlled physical/chemical/biological properties. Comparing to 3D printed monolthic scaffolds loaded with functional molecules for regenerating either cartilage tissue or bone tissue, multi-phasic scaffolds as well as scaffolds with gradient features have been developed via multi-material 3D printing or gradient 3D printing to mimic the heterogeneous features of native osteochondral tissue and regionally facilitate MSC morphogenesis along osteogenic or chondrogenic lineage [[10], [11], [12], [13]]. Furthermore, chondroinductive or osteoinductive biomolecules/drugs were loaded in the corresponding zone to further improve the chondrogenic/osteogenic differentiation of MSCs [14]. However, MSC-derived chondrocyte-like cells are more inclined to show a fibrochondrocyte phenotype, although chondrogenesis-related biomarkers such as sry related HMG box protein-9 (SOX9), collagen II and aggrecan were up-regulated.

From cell development perspective, the successful differentiation of MSCs into hyaline chondrocytes undergo several stages, including initial cell aggregation, tuning of cell morphology, initiation of chondrogenic differentiation, cell maturation and phenotype maintenance [15,16]. Among these steps, initial cell aggregation is of great importance as the initial cell morphology (round cell aggregates vs. discrete spreading cells) can determine whether these MSC can be differentiated into hyaline chondrocytes or fibrochondrocytes [17,18]. Therefore, the cartilage region of scaffoldsshould be endowed with excellent capability to tune the cellular morphology, allowing the formation of MSC aggregates to improve the chondrogenic differentiation [19,20]. Supramolecular hydrogels with dynamic bondings allow cells to migrate freely within the hyrogel matrices to achieve desirable cellular organizational structures via spontaneous aggregation [22]. However, as such hydrogels made of proteins or polysaccharides with varied hydrophilicity exhibit varied cell affinity and degradability, the long-term maintenance of the aggregated state of MSCs in the supramolecular hydrogel cannot be guaranteed. Therefore, functional biochemical agents such as TGF-β1/3 which are gold standard to induce MSC chondrogenic differentiation are still needed to achieve successful chondrogenic differentiation to hyaline chondrocytes. Since yes-associated protein (YAP) can modulate cell morphology by regulating the assembly and disassembly of F-actin, where YAP deactivation gradually drives cells toward a rounded phenotype accompanied by markedly reduced F-actin expression, incorporating YAP-inhibitory molecules in the cartilage zong of osteochondral scaffolds may also guide cells to obtain a desirable cellular organizational structure (i.e., round cell shape and cell aggregate) [23].

In this study, customized bi-phasic osteochondral scaffolds with heterogeneous features were fabricated through dual-material sequential cryogenic 3D printing, followed by dispensing of gelatin-based supramolecular hydrogel with dynamic bondings in the cartilage frame. The osteochondral scaffolds can adjust the initial cellular morphology of MSCs at respective region and facilitate their regional chondrogenic/osteogenic differentiation into hyaline chondrocytes and osteoblasts upon the regional delivery of TGF-β1 and osteogenic peptide (OP) at cartilage zone and subchondral zone, respectively, and eventually regenerate osteochondral tissue *in vivo*. This study provided a facile but versatile strategy to regenerate heterogeneous tissues via modulating and maintaining the cellular morphology of seed cells during tissue regeneration process.

## 2 Experimental

### 2.1 Materials

PLGA (LA:GA = 50:50, inherent viscosity of 0.76 dl g^−1^) and shape memory poly(D,L-lactide-co-trimethylene carbonate)‌ (P(DLLA-TMC)) (DLLA:TMC = 80:20, inherent viscosity of 0.78 dl g^−1^, transition temperature (Ttrans): 37 °C) were provided by Jinan Daigang Biotechnology Ltd, Jinan, Shandong, China. β-tricalcium phosphate (β-TCP) particles with an average diameter of 60 ± 10 μm were Bio-lu Limited Corporation products (Shanghai, China). Deionized (DI) water for all experiments was obtained using a DI water producer (Sartorius arium mini plus, Germany). Tween 20, phosphate buffered saline (PBS) tablets, bovine serum albumin (BSA), gelatin (from bovine), photoinitiator ‌lithium phenyl(2,4,6-trimethylbenzoyl)phosphinate (LAP) and TGF-β1 were SigmaAldrich products (USA). Dichloromethane (DCM) was supplied by Uni Chem Co., Korea. Poly ethylene glycol diacrylate (PEGDA) with a molecular weight of 5000 was Tansh-Tech product (China). Oesteogenic peptide (OP) with a sequence of KIPKA SSVPT ELSAI STLYL SGGC and a purity of 98.12 % was synthesized by Shanghai Ziyu Biotechnology Ltd, China. Acrylated-β-cyclodextrin (Ac-β-CD) was synthesized in our lab [24]. Polyethylene glycol diacrylate (PEGDA) with a Mw of 5,000 was supplied by GuangZhou Tanshtech Co., Ltd, China.

### 2.2 Formulation of printing inks

Towards the printing ink for fabricating subchondral frame, 1.5 ml of DI water was firstly blended with 10 ml of PLGA/DCM solution (20 %, w/v), 50 μl of Tween 20 and 4 g of β-TCP. After 5 min ultra-sonication, water-in-oil composite emulsion inks, consisting of continuous oil phase (PLGA/DCM), uniformly distributed β-TCP particles and discrete water phase (water droplet) were successfully formed. 500 μl of DI water containing 10 mg of OP and 20 mg of BSA (stabilizer) was then added into the water/β-TCP/PLGA/DCM emulsion, followed by 5 min of gentle manual mixing, hence forming the subchondral ink for printing the subchondral frame (designated as “Sub frame”). Towards the printing ink for fabricating cartilage frame in the cartilage layer, 0.5 ml of DI water was firstly blended with 5 ml of P(DLLA-TMC)/DCM solution (30 %, w/v) and 10 μl of Tween 20. After 5 min ultra-sonication, uniform water-in-oil emulsion ink for printing the cartilage pattern was formed. Then, 200 μl of DI water containing 10 μg of TGF-β1 and 2 mg of BSA (stabilizer) was then added into water/P(DLLA-TMC)/DCM emulsion, followed by 5 min of gentle manual stirring, hence forming cartilage ink for printing the cartilage frame (designated as “Cart frame”). Towards the supramolecular hydrogel ink for dispensing in the porous cartilage frame, 1.2 g of gelatin, 0.8 g of Ac-β-CD, 0.1 g of PEGDA and 10 mg of LAP were dissolved in 10 mL of DI water at 45 °C, followed by a 1 h magnetic stirring at 100 rpm to form supramolecular hydrogel precursor (designated as “Gel”).

### 2.3 Fabrication of osteochondral tissue engineering scaffolds

Osteochondral scaffolds with a disc shape were fabricated through a dual-nozzle multi-material 3D printing in a cryogenic environment (i.e. −20 °C). According to a pre-designed digital STL file with a column structure, firstly, 10 layers of subchondral struts (“OP/β-TCP/PLGA” strut) were printed to form the subchondral frame (about 3 mm in height, 0.3 mm each layer). Each layer of the subchondral frame consisted of 10 paralleled rods with a length between 2 and 10 mm and a diameter of 0.4 mm; the distance between two paralleled rods was 0.5 mm; rods in adjacent layers had a cross angle of 90°. After the 3D printing of the 10 layered subchondral frame, 2 more layers of subchondral struts which were parallel to the struts in the as-printed last layer were precisely deposited on the struts, to form a “concave-convex” structure with a 3-layer height. Afterwards, cartilage frame was 3D printed on the top of the as-printed subchondral frame. Firstly, 3 layers of cartilage struts (“TGF-β1/P(DLLA-TMC)” strut) with a parallel direction were printed to fill the gaps left in the “concave-convex” structure of the subchondral frame. Then, 6 layers of cartilage struts with a grid structure (about 1.2 mm in height, 0.2 mm each layer) were printed on the top of the as-printed paralleled cartilage struts. The as-printed osteochondral scaffolds consisting of both subchondral frame and cartilage frame were then subjected to a 10 h of freeze drying. Afterwards, supramolecular hydrogel precursor was dispensed in the pores of the cartilage frame, and 5 min of UV irradiation with a power of 100 mW/cm^2^ was conducted at 37 °C to crosslink the supramolecular hydrogel.

### 2.4 Scaffold characterization

The macroscopic morphology of scaffolds was examined using a digital camera (Huawei Mate 70 Pro+, Shenzhen, China) and a stereomicroscope (T6-HD208C, Aosvi Shenzhen Optical Instrument, Co., Ltd, China). The microstructure of scaffolds was observed using SEM (VeriosG4UC, Thermo Fisher, USA), in which scaffold samples were freeze dried, followed by coating of a thin layer of gold. The osteochondral scaffolds (Cart frame@Gel + Sub) as well as cartilage frames (Cart frame) and cartilage frames filled with supra gel (Cart@Gel) were subjected to compression testing under wet conditions at 37 °C. Five samples with a dimension of 10 mm × 10 mm × 10 mm were tested for each type of scaffold and the strain speed was set as 1 mm min ^−1^. The interfacial bonding strength between the subchondral layer and the cartilage layer of the osteochondral scaffolds was examined by shear testing and peel testing. Towards shear testing, lap shear ASTM D3163 testing is followed. The overlap length was 15 mm and the strain speed was set as 1 mm min ^−1^. Towards the peel testing, ASTMD3330 Method A (180° peel) was followed.

### 2.5 Cell culture

Rat bone marrow derived MSCs (rBMSCs) at P6 (provided by iCell Bioscience Inc., Shanghai, China) were cultured with iCell culture medium (iCell Bioscience Inc., Shanghai, China), and maintained in a humidified incubator at 37 °C with 5 % CO_2_. The medium was changed every 2 d. Once the cell achieved 80 % confluence, cells were treated with Trypsin-EDTA (Gibco, USA) to obtain cell suspension. Afterwards, 0.2 ml of rBMSC solution with a concentration 1 × 10^6^ cell ml^−1^ was blended with 0.2 mL of supramolecular hydrogel precursor and dispensed in the cartilage frame of the osteochondral scaffolds, followed by 5 min of UV crosslinking. Afterwards, the scaffold was inversely placed and the subchondral zone was seeded with 0.2 ml of rBMSC solution with a concentration 1 × 10^6^ cell ml^−1^.

### 2.6 Cell viability

After 1 and 7 d culture, a live and dead viability kit (Molecular Probes, USA) was used to stain rBMSCs in osteochondral scaffolds to visualize live and dead cells in the osteochondral scaffolds. For cell staining, cell-scaffold constructs were washed and incubated in iCell medium containing 4 μM EthD-1 and 2 μM calcein AM in a humidified incubator (37 °C, 5 % CO_2_) for 30 min, where live cells and dead cells were stained into green color and red color, respectively. Fluorescence images of cell-scaffold constructs were obtained using a fluorescence microscope (Nikon Eclipse TE2000-U inverted microscope, Japan). The cell proliferation at d 1 and d 7 was studied using CCK8 assay.

### 2.7 Chondrogenic/osteogenic differentiation of rBMSCs *in vitro*

After 14 d culture, rBMSC-laden osteochondral scaffolds were subjected to staining of nucleus (DAPI, Life Technologies), F-actin (GF 488 phalloidin, color in green, Guangzhou Gexion Biotechnology Co., Ltd, China), G-actin (Genmed Scientifics Inc., Shanghai, China), chondrogenic markers including SOX9, collagen II (COL II) and aggrecan (ACAN), osteogenic markers including Runt-related transcription factor 2 (RUNX2) and osteocalcin (OCN) (Boster Biological Technology co., Ltd, China) and Alexa Fluor(r) 555 Goat Anti-Rabbit IgG (H + L), (Life technologies, USA). The stained cell-scaffold constructs were then subjected to a confocal laser scanning microscope (CLSM, LSM 710 Meta, Carl Zeiss, Germany) for observations of nucleus, F-actin, G-actin and chondrogenic/osteogenic markers in both subchondral layer and the cartilage layer.

### 2.8 Animal experiments

The intravenous anesthesia were anesthetized by administering 10 mL of ethyl carbamate (0.2 g/mL) via the ear vein of the rabbits. After confirming the effectiveness of anesthesia, knee arthrotomy was performed through a medial parapatellar incision. Then the patella was everted to reveal the femoral articulation. A 4 mm in diameter and 5 mm in depth circular osteochondral defect was made using a trephine. The actual position of the defect was oriented at the center point of the femoral trochlear (the intersection point of the longitudinal axis trochlear groove and midline through medial and lateral condyles). Scaffolds were subsequently implanted into each defect. After the construction of the defected osteochondral model in rabbit femoral Trochlea, different groups of cylindrical osteochondral scaffolds (with or without (w/o) growth factor delivery) (diameter: 4 mm; height of subchondral zone: 10 mm; height of cartilage zone: 1.5 mm) were implanted in defected cavities and blood containing bone marrow derived MSCs was diffused into the pores of the osteochondral scaffolds. Negative control group leaving only defected cavities which was filled by the blood containing bone marrow derived MSCs was also including in the animal tests. 3 months after implantation, all rabbits were sacrificed by CO_2_ and the specimens were taken out and fixed with PFA (4%) to conduct MRI imaging and μ-CT imaging. Afterwards, specimens were treated with EDTA solution for 3 months of decalcification. Then the samples were subjected to paraffine embedding and tissue slicing. The sliced samples were subsequently subjected to 5 types of histochemical staining, including H&E staining, Masson's trichrome staining, Safranin O-fast green (SO-FG) staining, Alician blue staining and Toluidine blue staining to examine the regeneration extent of the osteochondral tissue in different groups.

### 2.9 Statistical analysis

All *in vitro* experiments included at least three replicates for each sample (n = 3), while *in vivo* experiments included at least five replicates for each group (n = 5), and numerical data are expressed as mean ± standard deviation. The level of statistical significance was determined by a one-way or two-way analysis of variance (ANOVA) with the Bonferroni post-hoc test (Graph Pad Software 9, Inc.). A difference of *p*∗ < 0.05 was considered to be significant.

## 3 Results

### 3.1 Design of osteochondral scaffold

**Fig. 1:**
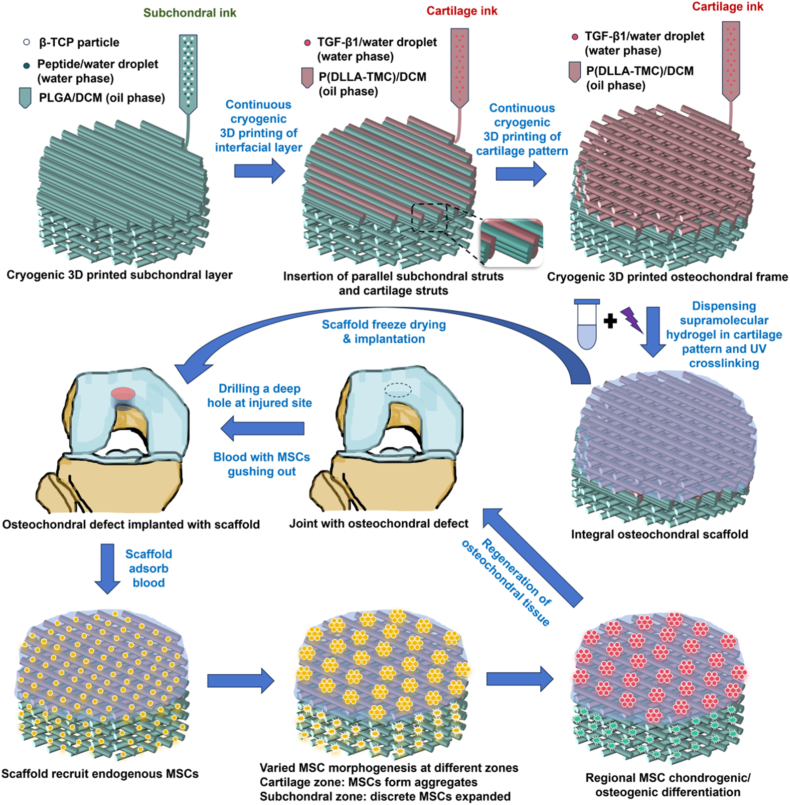
Schematic illustration of the fabrication process of osteochondral scaffolds, the evolution of cellular organizational structure of MSCs in different regions of the osteochondral scaffolds, and the regeneration of defected osteochondral tissue *in vivo*.

In this study, an advanced heterogeneous structured osteochondral scaffold loaded with chondrogenic/osteogenic growth factors at different regions was designed and fabricated through dual-material sequential cryogenic 3D printing of the subchondral frame and cartilage frame, followed by the dispensing of a supramolecular hydrogel in the cartilage frame. Given that cells can migrate freely in the double-network dynamically crosslinked supramolecular hydrogel, such scaffold had the capability to spatially tune the initial cellular organizational structure of MSCs, leaving aggregated MSC microspheres and discrete spindle-shaped MSCs in the cartilage zone and subchondral zone, respectively, which were favorable for simultaneous chondrogenic/osteogenic differentiation and integrated regeneration of osteochondral tissue. However, the sole use of such osteochondral scaffold cannot ensure the successful differentiation of MSCs into targeting cells, especially the chondrogenic differentiation of MSC aggregates with a spherical shape into hyaline chondrocyte clusters. Therefore, inductive growth factors such as TGF-β1 and OP should be loaded in the scaffold as well to ensure the efficient differentiation and hence led to successful integrated regeneration of the defected osteochondral tissue. Fig. 1 shows the fabrication process of the osteochondral scaffold and the process of treating osteochondral defects using such scaffold.

### 3.2 Physical characterization of osteochondral scaffolds

**Fig. 2:**
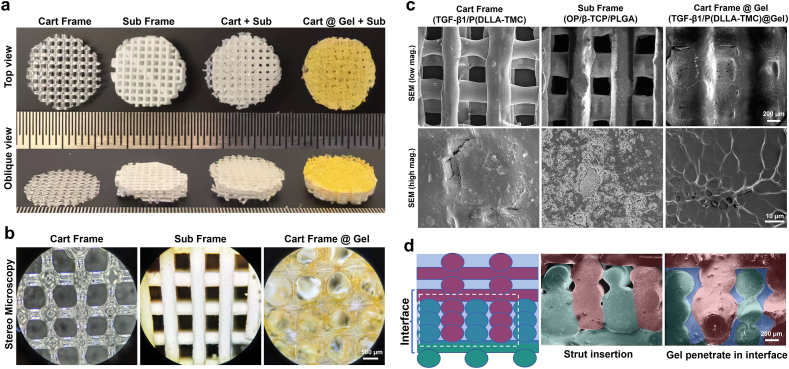
Morphology of cryogenic 3D printed osteochondral scaffolds with heterogeneous structure: (a) top view and oblique view of cartilage frame, subchondral frame, osteochondral frame and osteochondral frame dispensed with supramolecular hydrogel in the cartilage frame; (b) stereo microscopic images of cartilage frame, subchondral frame and cartilage frame filled with hydrogel; (c) SEM micrographs of cartilage frame, subchondral frame and cartilage frame filled with hydrogel, with varied magnification; (d) designed and actual structures of osteochondral interface consisting of alternately deposited cartilage struts (color in red) and subchondral struts (color in green), and hydrogel (color in blue). (For interpretation of the references to color in this figure legend, the reader is referred to the Web version of this article.)

Fig. 2a shows the macroscopic morphology of cartilage frame (Cart frame), subchondral frame (Sub frame), integrated osteochondral frame (Cart frame + Sub frame) and integrated osteochondral scaffold (i.e., Cart frame@Gel + Sub frame) (top view and oblique view). The subchondral scaffold had a disk-like shape with a diameter of 10 mm and a height of 3 mm while the cartilage frame had a disc-like structure with a diameter of 10 mm and a height of 1.5 mm. Magnified macroscopic morphology of osteochondral scaffolds was examined using stereomicroscopy (Fig. 2b). Each layer of the transparent grid structured cartilage frame consisted of paralleled struts with an average diameter of 420 ± 40 μm and a spacing distance of 500 ± 50 μm, and the struts in adjacent layers had a cross angle of 90°. The struts in the subchondral frame (color in white) had similar structural parameters, showing paralleled struts with an average diameter of 400 ± 25 μm and spacing distance of 500 ± 40 μm. The supramolecular hydrogel was filled in the square pores of the cartilage frame (color in bright yellow). Fig. 2c shows the SEM micrographs of osteochondral scaffolds with varied magnifications. As shown in the SEM micrographs with a low mag., both cartilage frame and subchondral frame exhibited grid-like porous structure while the cartilage frame was filled with supramolecular hydrogel. The SEM micrographs with a high mag. exhibited detailed features of struts in the cartilage frame and subchondral frame and β-TCP particles can be observed in the PLGA matrix of the subchondral frame. The magnified image of supramolecular hydrogel exhibited a typical freeze dried hydrogel morphology. Fig. 2d shows the designed and actual cross-sectional structures of the osteochondral interface, where subchondral struts (color in dark green) and cartilage struts (color in dark red) were alternately deposited to form an insertion structure. Fusion of adjacent struts at the horizontal direction was also observed, which could contribute to the high interfacial bonding strength. After the dispensing of supramolecular hydrogel in the cartilage frame, some hydrogel (color in dark blue) penetrated into the interface region and filled the cavities left in the osteochondral interface.

**Fig. 3:**
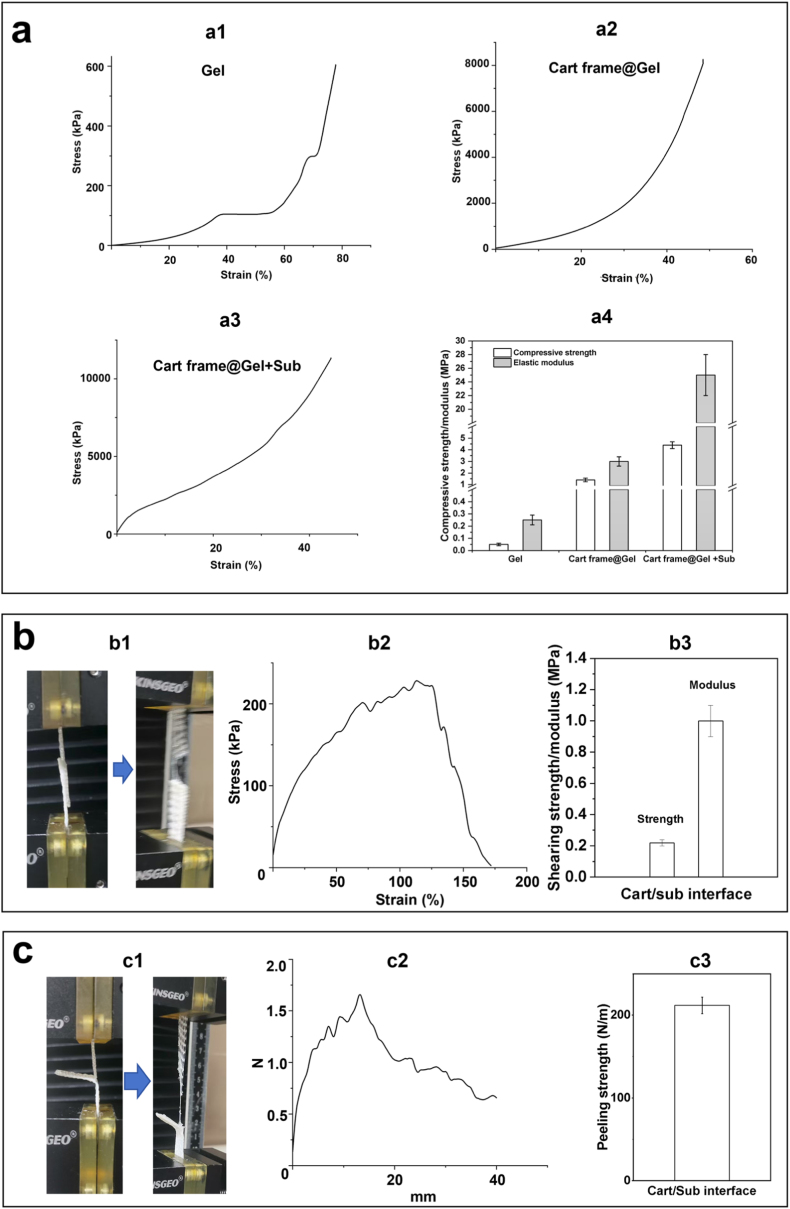
Mechanical properties of osteochondral scaffolds and control groups (n = 5): (a) compressive strengths/moduli and respective stress-strain curves; (b) shear testing, stress-strain curve and shear strength/modulus between cartilage layer and subchondral layer; (c) peeling testing, typical peeling testing curve and peeling strength between cartilage layer and subchondral layer.

The overall mechanical property of the osteochondral scaffolds was evaluated using compression testing at 37 °C, while shear testing and peeling testing were employed to study the interfacial bonding strengths. Fig. 3a shows the typical stress-strain curves of supramolecular hydrogel (“Gel”, Fig. 3a1), cartilage frame filled with supramolecular hydrogel (“Cart frame@Gel”, Fig. 3a2) and the whole osteochondral scaffolds (“Cart@Gel + Sub”, Fig. 3a3), and the compressive strengths and elastic moduli (Fig. 3a4). By collecting compressive strength data at a strain of 20 %, the compressive strengths of different groups showed significant difference (*p* < 0.05), where “Gel” group had the lowest value (0.05 MPa) and “Cart@Gel + Sub” group had the highest value (5 MPa). The “Cart frame@Gel” group had a compressive strength of 1.5 MPa. Similarly, the moduli of these groups also exhibited significant difference (*p* < 0.05), and the compressive modulus of Cart frame@Gel group was still comparable to that of the native cartilage tissue (3.5 MPa vs. 0.5–2 MPa (instant), 5–15 MPa (balanced). The osteochondral interface had a shear strength and an elastic modulus of 0.22 MPa and 1.0 MPa, respectively (Fig. 3b2 and 3b3), which are similar to that of native osteochondral interface (strength: 0.25–0.7 MPa; modulus: 0.5–1.5 MPa). The peeling strength (peeling force/width) between the cartilage layer and the subchondral layer was 212 N/m (Fig. 3c2 and 3c3), which also falls in the peeling strength range of the native osteochondral tissue (100–300 N/m). It is worth noting that, the fracture of the shear testing samples and peeling testing samples occurred at the elastic shape memory cartilage region (not at the central part of the sample) (Fig. 3b1 and 3c1), which had a lower tensile strength than the subchondral layer and the closely bonded cartilage/subchondral interface, suggesting that the actual shear strength and peeling strength between the cartilage layer and the subchondral layer could be higher than that collected by the testing machine.

### 3.3 Biological performance of osteochondral scaffolds

**Fig. 4:**
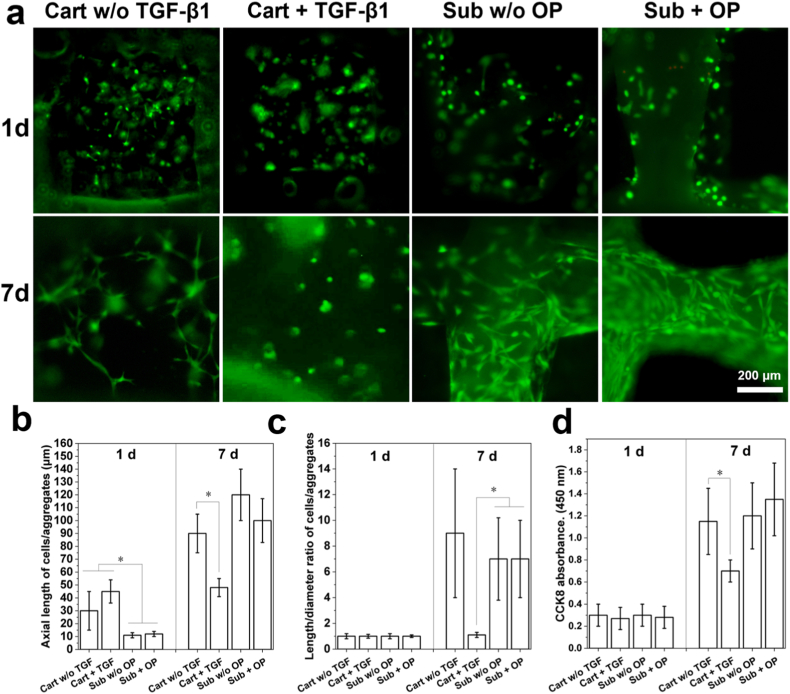
Viability of rBMSCs seeded in osteochondral scaffolds with and without (w/o) delivery of TGF-β1 and OP in respective zone: (a) live and dead staining of MSCs cultured in different osteochondral scaffolds for 1 and 7 d; (b) axial length of cell/cell aggregates in different osteochondral scaffolds with varied culture time; (c) length/diameter ratio of cell/cell aggregates in different osteochondral scaffolds with varied culture time; (d) CCK8 absorbance of MSCs cultured in different osteochondral scaffolds at day 1 and 7 (n = 3).

Biological performance of the osteochondral scaffolds was then studied by investigating the cell viability on scaffolds through live and dead staining and CCK8 assay. As shown in Fig. 4a, after 1 d culture, most MSCs seeded in the subchondral zone were alive and found to attach to the subchondral strut surface. Some MSCs showed an expanded shape while the rest of them exhibited a round shape, no matter OP was loaded in the struts or not. In comparison, nearly all MSCs seeded in the hydrogel bricks at the cartilage zone were alive and many of them aggregated spontaneously to form MSC microspheres with a larger diameter (*p* < 0.05) (Fig. 4b). Interestingly, it is found that a small part of MSCs located in the cartilage zone w/o the incorporation of TGF-β1 had an expanded shape by showing elongated cell body. In comparison, no cell expansion was found in MSCs located in the cartilage zone incorporated with TGF-β1. After 7 d culture, all MSCs seeded on subchondral struts exhibited spindle-like morphology, no matter OP was loaded in struts or not, and subchondral zone loaded with OP induced higher level of cell density. Towards MSCs in hydrogel blocks at the cartilage zone, elongated cells with a slender shape were observed when no TGF-β1 was loaded, showing a very high length-to-diameter (L/D) ratio (Fig. 4c). In contrast, when TGF-β1 was loaded in the hydrogel bricks at the cartilage zone, cells still exhibited an aggregated state (L/D = 1) and no cell elongation was observed. The proliferation of MSCs in different zones with or w/o growth factor loading is shown in Fig. 4d. Obvious cell proliferation was detected in MSCs seeded in cartilage zone from d 1 to d 7 when no TGF-β1 was loaded, whereas much slower cell proliferation was detected for MSCs seeded in cartilage zone loaded with TGF-β1 (*p* < 0.05). In comparison, MSCs seeded in subchondral zone either loaded with OP or not showed distinct cell proliferation and OP induced higher cell proliferation rate.

### 3.4 *In vitro* chondrogenic/osteogenic differentiation of MSCs in respective zone

**Fig. 5:**
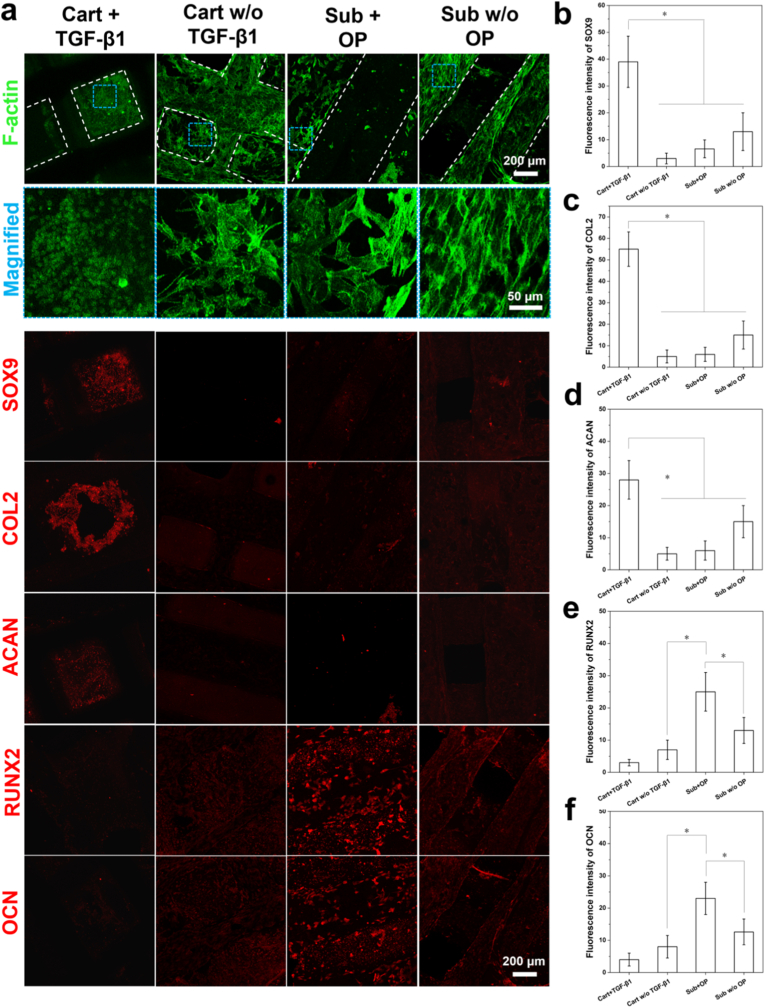
(a) Immunofluorescence images of rBMSCs stained with F-actin (color in green), chondrogenic markers (SOX9, COL2, ACAN) and osteogenic markers (RUNX2, OCN) in different zones of the osteochondral scaffolds, with or w/o the delivery of growth factors after 14 d culture; (b–f) Histograms of fluorescence intensity of different biomarkers expressed in MSCs located at different zones of the osteochondral scaffolds (n = 3) (∗p < 0.05). (For interpretation of the references to color in this figure legend, the reader is referred to the Web version of this article.)

As shown in Fig. 5a, after 14 d culture, when TGF-β1 was loaded in the cartilage zone, cells seeded in the hydrogel bricks maintained their round shape and stayed at their initial sites, and no F-actin filament extension was observed. These cells expressed a high level of chondrogenic biomarkers such as SOX9, COL II and ACAN, and a very low level of osteogenic biomarkers such as RUNX2 and OCN (Fig. 5b–f). In comparison, when no TGF-β1 was loaded in the cartilage zone, some cells laden in the hydrogel bricks broke through the hydrogel and migrated to the cartilage frame. Besides, both cells left in the hydrogel bricks and cells migrated to the strut surface exhibited an expanded cell shape and distinct F-actin filament extension can be observed. Moreover, these cells exhibited a much lower level expression of SOX9, COL II and ACAN (*p* < 0.05), and slightly up-regulated expression of RUNX2 and OCN was observed. Towards cells in the subchondral zone, when OP was loaded, a very low level of expression of SOX9, COL II and ACAN was detected in the polygonal shaped cells spreading on the strut surface while a significantly higher level of expression of RUNX2 and OCN was observed (*p* < 0.05). Likewise, when OP was not loaded in the subchondral zone, the chondrogenic markers (i.e., SOX9, CLO II and ACAN) was slightly up-regulated in the spindle-shaped cells with numerous elongated F-actin filaments while the expression of RUNX2 and OCN was down-regulated (*p* < 0.05).

**Fig. 6:**
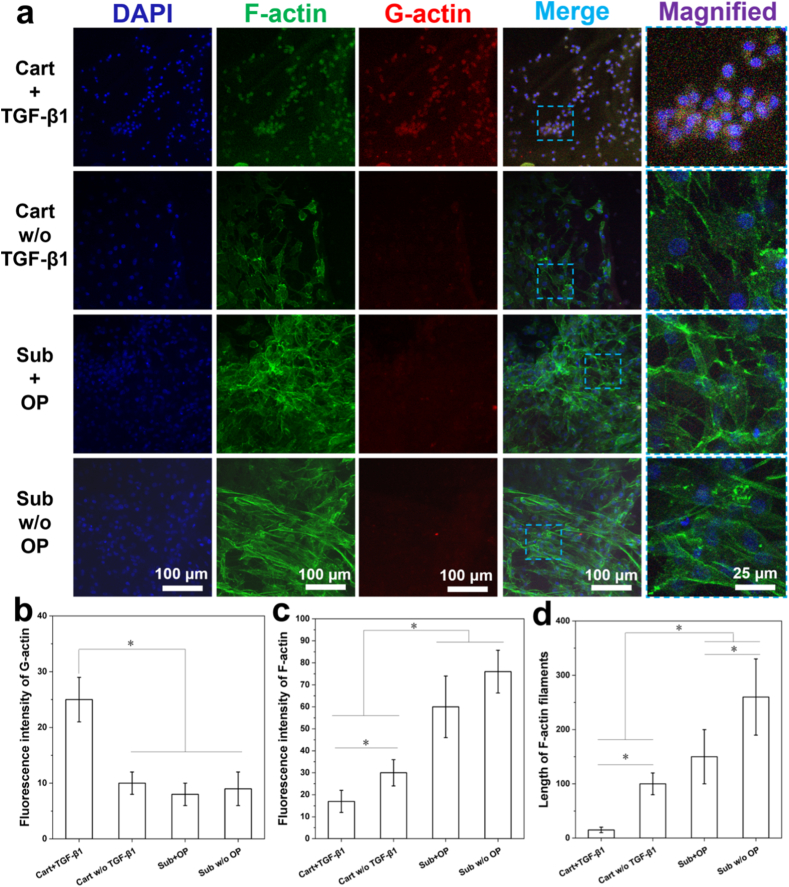
(a) Immunofluorescence images of rBMSCs stained with nucleus (color in blue) F-actin (color in green) and G-actin (color in red) in different zones of the osteochondral scaffolds, with or w/o the delivery of growth factors after 14 days of culture; (b–c) fluorescence intensity of G-actin and F-actin expressed in cells; (d) Length of F-actin filaments in cells cultured in different zones of the osteochondral scaffolds, with or w/o the delivery of growth factors (n = 3). (For interpretation of the references to color in this figure legend, the reader is referred to the Web version of this article.)

The actin expression of cells cultured in different groups for 14 d was also studied by visualizing both the polymerized F-actin and G-actin monomer (Fig. 6a). When TGF-β1 was loaded, cells in the cartilage zone exhibited a low level of F-actin expression and a high level of G-actin expression, in terms of fluorescence intensity (Fig. 6b and c) (*p* < 0.05), suggesting that during the efficient chondrogenic differentiation of MSCs which was induced by TGF-β1, the actin polymerization was inhibited and most actin molecules were maintained at the monomer state. In comparison, when no TGF-β1 was loaded, polygonal cells in the cartilage zone showed up-regulated F-actin expression in terms of both fluorescence intensity (Fig. 6c) and filament length (Fig. 6d) (*p* < 0.05), whereas a very limited G-actin expression was observed. Cells in the subchondral zone loaded with OP or not showed similar trends, where more distinct F-actin expression was accompanied by much less G-actin expression (*p* < 0.05).

### 3.5 *In vivo* osteochondral tissue regeneration

**Fig. 7:**
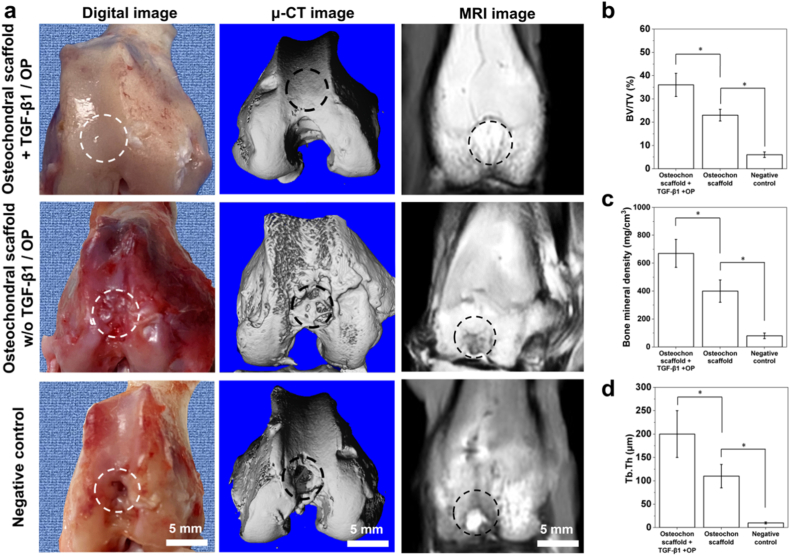
Imaging analysis of tissue regenerated by different groups of osteochondral scaffolds (n = 5): (a) general images, μ-CT images and MRI images of rabbit osteochondral defects treated with different groups of repairing materials including osteochondral scaffolds loaded with growth factor, w/o growth factors and negative control group, 3 month post-surgery, White dash circles and black dash circles indicate the defected site; (b) BV/TV ratio of regenerated subchondral tissues; (c) bone mineral density (BMD) of regenerated subchondral tissues; (d) trabecula thickness (Tb. Th) of regenerated subchondral tissues.

*In vivo* animal studies were then conducted to verify the effectiveness of our advanced osteochondral scaffolds. Macroscopic morphology of rabbit femoral trochlea implanted with osteochondral scaffolds was captured by a digital camera, while bone formation and cartilage regeneration were investigated by conductingμ-CT imaging and MRI imaging. As shown in the digital images of Fig. 7, the tissue regenerated by osteochondral scaffolds loaded with TGF-β1 and OP had a smooth surface which was identical to the peripheral host cartilage tissue, whereas the tissue regenerated by osteochondral scaffolds w/o TGF-β1/OP loading had a relatively rough surface with a concave and convex structure. In comparison, a cavity still can be found in the central part of the defect in the negative control group and slight tissue regeneration can be observed at the peripheral part of the defect. It can be seen from the μ-CT images of Fig. 7a that excellent subchondral tissue regeneration was also observed 3 month after the implantation of osteochondral scaffold loaded with TGF-β1/OP. The subchondral bone tissue was dense and no micropores were found in the regenerated site. In comparison, imperfect bone regeneration was obtained for the osteochondral scaffold group w/o TGF-β1/OP loading and several small cavities can be observed, whereas the negative control group had the worst bone forming situation by showing a huge cavity in the central site of the regenerated bone tissue. The regenerated subchondral tissue induced by the osteochondral scaffolds loaded with TGF-β1/OP had significantly higher bone volume/total volume (BV/TV) ratio, bone mineral density (BMD) and trabecula thickness (Tb.Th) than that induced by osteochondral scaffolds w/o TGF-β1/OP loading, and the negative control group showed the lowest level of BV/TV, BMD and Tb.Th (Fig. 7b, c and d) (*p* < 0.05). It can be also seen from MRI images that osteochondral scaffolds loaded with TGF-β1 and OP induced excellent cartilage regeneration, since no white dot area was found. In comparison, gray area with different shades and bright white light spot were observed in osteochondral scaffold group and the negative control group, respectively, indicating the imperfection cartilage regeneration and failed osteochondral regeneration in these two groups, respectively.

**Fig. 8:**
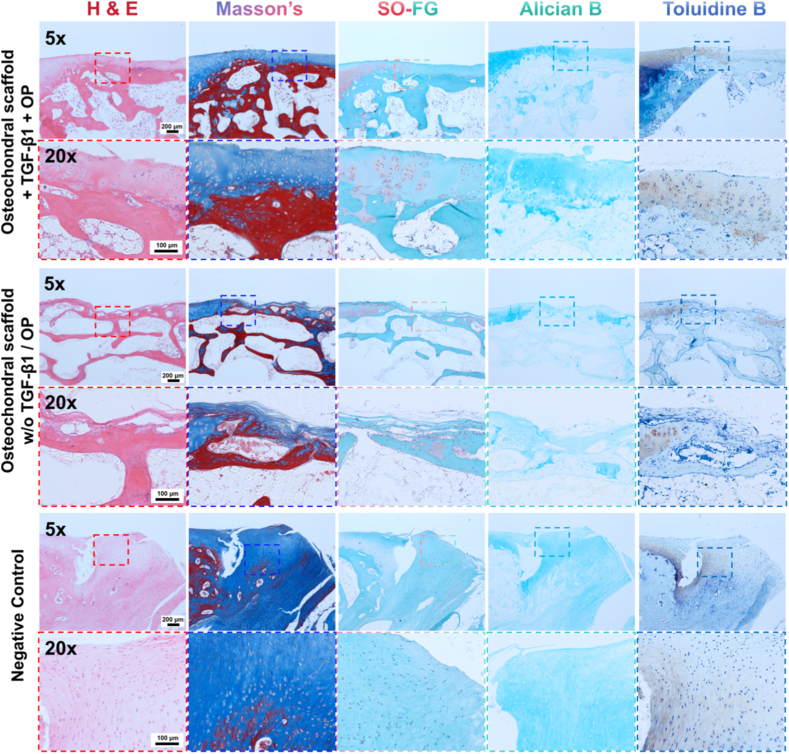
Optical microscopic images of tissue slices of 3 groups (osteochondral scaffolds with growth factor group, osteochondral scaffolds w/o growth factor group and negative control group, n = 5) of regenerated osteochondral tissue 3 month post-implantation, which were subjected to H&E staining, Masson's trichrome staining, Safranin O-Fast green staining, Alician blue staining and Toluidine blue staining, including 5x images showed the over view of the regenerated tissue while 20x images showed the detailed features of selected area. (For interpretation of the references to color in this figure legend, the reader is referred to the Web version of this article.)

Tissue slicing and 5 types of histochemical staining including H&E, Masson's trichrome, SO-FG, Alician blue and Toluidine blue staining were then conducted to examine the histology of the regenerated osteochondral tissues (Fig. 8). As shown in the Negative control group, only highly aligned fibrocartilage tissue can be found in the defect. This could be attributed to the formation of blood clots in the defect after bone marrow stimulation surgery, which normally induced to the formation of fibrocartilage tissue. When osteochondral scaffolds w/o TGF-β1/OP delivery were implanted in the defects, subchondral tissue regeneration with a certain extent was observed, however, only very thin regenerated cartilage tissue with a discontinuous state can be found, where no matured hyaline chondrocytes can be found. Likewise, trebecular structure with a low trebecular bone density and thin diameter (80–125 μm) can be found in the subchondral zone. Differently, when osteochondral scaffolds loaded with TGF-β1/OP were implanted, significantly improved osteochondral tissue regeneration with classic osteochondral structure, comprising of clearly visible cartilage zone, interfacial layer and subchondral zone, was obtained in the defected sites. The thickness of the regenerated hyaline cartilage was larger than 600 μm, which was significantly larger than that in the osteochondral scaffold alone group (*p* < 0.05). The subchondral bone exhibited a higher maturity by showing thick trebecular structure (trebecula diameter range: 133–250 μm, color in dark red in the Masson's trichrome image). Vertically aligned hyaline chondrocytes with a round shape were also found in the regenerated cartilage tissue and matured cartilage lacuna (color in pink in the SO-FG images) gradually extended from the part near the host tissue to the central part of the regenerated cartilage tissue. Similarly, Alician B image and Toluidine B image of this group also confirmed successful regeneration of the ostecohondral tissue where specific staining colors gradually extended from the regenerated tissue near to the host tissue to the central part of the regenerated cartilage tissue.

## 4 Discussion

So far, a variety types of osteochondral scaffolds have been fabricated to achieve integrated osteochondral regeneration [[25], [26], [27]]. Among these, scaffolds with gradient features which are made through multi-material 3D printing has gained increasing attention as them can regionally mimic the structural and compositional features of the natural osteochondral tissue, and improve MSC osteogenic/chondrogenic differentiation spatially under the induction of drugs/biomolecules loaded at respective regions [28]. Researchers also tried to provide 3D printed scaffolds with biomimetic mechanical strengths by using polyesters and hydrogels to construct the subchondral layer and cartilage layer [29]. However, as hydrogels and polyesters such as PCL have varied hydrophilicity, sufficient interfacial bonding strength cannot be guaranteed. Therefore, biodegradable polyesters with varied elastic properties can be used as desirable printing inks to fabricate both cartilage frame and subchondral frame with high bonding strength, where hydrogels are filled in the porous cartilage frame to provide ECM-like environment for chondrocytes/MSCs, while subchondral frame containing bioceramic particles can be directly used as the trebecula-like bony environment to induce osteogenic differentiation of MSCs [30]. In our previous study, polyesters with varied elastic modulus were used as matrices of printing inks to fabricate the osteochondral frames with varied regional strengths. However, as the bottom of the cartilage layer was directly printed on the top of the subchondral layer, the contact area was not large enough to provide excellent interfacial bonding strength [14]. Inspired by Tetris game, in this study, the bottom struts of the cartilage frame and the top struts of the subchondral frame were designed as complementary comb structures to enable mutual insertion and a dense osteochondral interface comprising of alternately deposited cartilage struts and subchondral struts can be formed. As the distance among the as-printed paralleled struts was very narrow, fusion among these struts would frequently occur during the solidification process (i.e., drying), hence leading to a higher interfacial bonding strength. In addition, as the temperature-sensitive shape memory cartilage frame became soft at 37 °C while the subchondral frame was still rigid at 37 °C, the heterogeneous mechanical features of the native osteochondal tissue can be simulated. Besides fabricating osteochondral frames, filling hydrogels in the cartilage zone is also an important step to provide seed cells with a favorable microenvironment for cell anchoraging, proliferation, morphogenesis and differentiation. In our previous study, collagen I hydrogel was employed to fill the cartilage frame. Although excellent cell adhesion and improved cell proliferation were obtained, such cartilage zone lacked of considerations about the effect of cellular environment on cell morphogenesis, especially on the efficiency of MSC chondrogenic differentiation into hyaline chondrocytes, resulting in the formation of a large amount of fibrochondrocytes in the cartilage zone [14].

The differentiation of MSCs into hyaline chondrocytes is a highly programmed physiological process involving gene cascade regulation, cell morphological remodeling, and ECM specific deposition. At 0–24 h, damaged or developing cartilage tissue releases TGF-β superfamily factors (TGF-β 1/3, BMP-2/7) and stromal cell-derived factor such as SDF-1 to guide MSC migration towards cartilage injury areas. Discrete MSCs then form MSC aggregates (also known as pre-chondrogenic condensation) via the adhesive effect of N-cadherin, to provide MSCs with favorable hypoxia chondrogenic differentiation environment [31]. Previous studies normally adopt chemically crosslinked hydrogels as the ECM-like environment to load MSCs, hence MSCs evenly distributed in such hydrogel blocks (no matter *in situ* laden in hydrogel or post seeded in freeze dried hydrogel) cannot alter their spatial distribution afterwards, restricting their spontaneous aggregation and hence resulting in low chondrogenic differentiation efficiency to hyaline chondrocytes and even the formation of fibrochondrocytes [14,18]. In our previous study, MSC aggregates and discrete MSCs were seeded simultaneously at the cartilage zone (i.e., P(DLLA-TMC) frame filled with GelMA hydrogel containing FGF-18) and subchondral zone (i.e., TCP/PLGA frame containing BMP-2), respectively [32]. Significantly improved chondrogenic/osteogenic differentiation of MSCs at the cartilage zone/subchondral zone into hyaline chondrocytes/osteoblasts were achieved. Cao *et al.* found that MSC aggregates had much better chondrogenic differentiation capability than discrete MSCs and the addition of TGF-β boosted this trend and eventually achieved desirable chondrogenic differentiation outcomes [33]. These studies confirmed that MSC aggregates were superior cell source than discrete MSCs to achieve desirable chondrogenic differentiation. However, preparing MSC aggregates and seeding such MSC aggregates in the cartilage zone of osteochondal scaffolds are relatively complicated and an even distribution of MSC aggregates at the cartilage zone was not easy to achieve. Therefore, smart hydrogels which allow seed cells to migrate freely should be used to fill the cartilage frame to achieve spontaneous MSC aggregation.

Double crosslinked supramolecular hydrogels with both chemical bonding and reversible “host-guest” complexation bonding provide cells with a favorable ECM-like environment to freely migrate and aggregate. The physical bonding between the aromatic structure in gelatin and bowl-like structure in β-CD can be broken when cells go through and get recovered via “host-guest” complexation when cells leave the site. Therefore, in the current study, the use of such supramolecular hydrogels instead of conventional UV crosslinked hydrogels such as GelMA provided discretely seeded rBMSCs with a dynamic ECM-like environment, allowing the rapid initial MSC aggregation in 1 d, and obtaining an aggregated cellular organizational structure which was favorable for better chondrogenic differentiation into hyaline chondrocytes. Moreover, the delivery of TGF-β1 in the cartilage zone enhanced such cell aggregation process by showing MSC aggregates with a larger average diameter, and this could be attributed to the fact that TGF-β signaling has the capability to guide the migration of MSCs for aggregation [34]. When longer culture time (i.e., 7 d) was reached, cell aggregates with and w/o TGF-β1 delivery showed significantly varied cellular organizational structure (spherical shape vs. fibroblast-like shape with extended F-actin filaments). Likewise, after 14 d culture, cell aggregates and discrete cells in the cartilage zone with the presence of TGF-β1 continuously maintained their spherical shape which was similar to that of the hyaline chondrocytes, whereas cells in the cartilage zone w/o TGF-β1 delivery exhibited fibrochondrocyte-like morphology. Moreover, the cartilage zone loaded with TGF-β1 induced much better chondrogenic differentiation than the cartilage zone w/o TGF-β1 loading, indicating that TGF-β1 had a dual function during the chondrogenic differentiation of MSC aggregates into hyaline chondrocytes: (1) quickly launch the chondrogenic differentiation of MSCs; and (2) long-term maintenance of the cellular organizational structure of cells at the aggregated state by suppressing the F-actin polymerization (as shown in Fig. 5) [35]. The above two effects eventually contributed to the differentiation of MSCs into hyaline chondrocyte-like cell clusters.

On the contrary, when no TGF-β1 was involved, neither maintenance of the spherical shape nor improved chondrogenic differentiation of MSCs can be achieved. Such huge difference on the cellular organizational structures can be attributed to following reasons: (1) MSCs w/o TGF-β1 induction can continuously secret matrix metalloproteinase (MMP) such as MMP-2, MMP-3, and MMP-13 to degrade surrounding gelatin-based hydrogel, hence producing spaces for F-actin filament extension; and (2) MSCs w/o TGF-β1 induction surrounded by the gelatin-based supramolecular hydrogel had a natural tendency to allow cell elongation and expansion during long-term culture, since gelatin which has a high cell affinity and favorable hydrophilicity for excellent cell adhesion was used as a key component of the supramolecular hydrogel. Therefore, strategies such as suppressing F-actin polymerization level by using inhibitory biochemical molecules and inhibiting F-actin filament extension via spatial physical constraints could be adopted to maintain the aggregated cellular organizational structure and hence improve chondrogenic differentiation.

On one hand, as YAP affects the stability of the cytoskeleton and morphological changes of cells by regulating the assembly and dissociation of F-actin, the antagonists of YAP and other closely related molecular targets such as fascin actin-bundling protein 1 (FSCN1) which can suppress the F-actin expression level can be used as potential drugs to replace TGF-β1/3 for efficient MSC chondrogenic differentiation into hylaine chondrocytes and maintain the cellular organizational structure of cells during differentiation [36,37]. On the other hand, anti-adhesive polysaccharide hydrogel with lower sensitivity to MMPs could be employed as hydrogel matrix to replace protein-based hydrogel to maintain the spherical structure of the MSC aggregates and improve chondrogenic differentiation into hyaline chondrocytes.

In the current study, the *in vivo* imaging data (i.e., digital images, μ-CT images and MRI images) and related imaging analysis (i.e., BV/TV, BMD and Tb.Th) suggest that the subchondral zone w/o OP loading cannot well regenerate the osteochondral tissue since a bony environment (i.e., TCP/PLGA struts) alone was insufficient to induce desirable osteogenesis. Therefore, OP was required to enhance subchondral bone formation. Meanwhile, chondrogenic growth factors such as TGF-β1 was required in the cartilage zone to achieve long-term maintenance of the organizational structure of cell aggregates, and hence improve the formation of continuous hyaline cartilage tissue on the top of the subchondral tissue. The *in vivo* histochemical results further confirmed that the combined used of supramolecular hydrogel which enabled spontaneous formation of MSC aggregates and the sustained release of TGF-β1 can guarantee the excellent regeneration of cartilage zone of defected osteochondral tissue by showing much improved cartilage regeneration where vertically aligned hyaline chondrocytes as well as matured cartilaginous lacuna can be found, and this was consistent to the *in vitro* results. Likewise, subchondral frame loaded with OP significantly enhanced subchondral bone regeneration by showing thicker trebecular diameter and darker staining shade in the Masson's trichrome image, which was also consistent to the *in vitro* results.

Despite its promising results, this study still has limitations. Firstly, the protein-based supramolecular hydrogel matrix used in this study has high cell affinity and is easy to be degraded by MMPs secreted by MSCs, eventually resulting in cellular fibrosis due to the over expressed F-actin. Therefore, supramolecular hydrogels which are not sensitive to MMPs should be developed to replace current protein-based hydrogel. Secondly, although assumptions about how to maintain the cellular organizational structure of cell aggregates in a long-term manner and how to enhance their chondrogenic differentiation into hyaline chondrocytes w/o the involvement of TGF-β have been proposed, experiments should be done to verify such hypothesis. Third, better strategy should be adopted to control and homogenize the size of the cell aggregates to better mimic the spatial distribution of hyaline chondrocytes in the middle-deep zone of the native cartilage layer.

## 5 Conclusion

In this study, bi-phasic osteochondral scaffolds with a heterogeneous structure and the ability to tune the cellular organizational structure of MSCs was successfully made through multi-material sequential cryogenic 3D printing of OP/β-TCP/PLGA subchondral frame and TGF-β1/P(DLLA-TMC) cartilage frame, followed by the dispensing of gelatin-based supramolecular hydrogel in the cartilage frame. When MSCs were seeded in different zones of the ostechondral scaffolds *in vitro*, differed cell morphogenesis routes were obtained by showing discretely distributed MSCs with an expanded morphology in the subchondral zone and spontaneously formed MSC aggregates in the cartilage zone. However,the MSC aggregates in the cartilage zone would lose their clustered structure and obtain a slender cell shape within 7 d and eventually differentiate into fibrochondrocyte-like cells if no TGF-β1 was delivered. When TGF-β1 was mediated, MSC aggregates were efficiently differentiated into hyaline chondrocytes by showing significantly up-regulated chondrogenic biomarker expressions. The OP delivered in the subchondral zone also improved the MSC osteogenic differentiation in this region. The *in vivo* animal study confirmed that growth factor-loaded osteochondral scaffolds can successfully regenerate defect osteochondral tissue, where TGF-β1 and supramolecular hydrogel which can induce spontaneous MSC aggregate formation had a synergistic effect on regenerating matured hyaline cartilage formation. In comparison, when no growth factors were loaded in the scaffolds, failure of hyaline cartilage regeneration would occur and the subchondral bone can only be regenerated to some degree. Such customized osteochondral scaffold also shows great translational potential due to its capacity to replicate native tissue heterogeneity. Its modular design can be readily adapted for different bioactive cues or cell types, enabling personalized strategies for treating different types of osteochondral defects. Future work can focus on the replacement of current smart hydrogel with FDA approved hydrogel having similar cellular responsiveness, and production, functional evaluation and *in vivo* validation of osteochondral scaffolds loaded with biomolecules/drugs approved by FDA which are functionally similar to TGF-β1/OP, to achieve integrated osteochondral regeneration and hence facilitate clinical translation.

## CRediT authorship contribution statement

**Lu Bai:** Writing – original draft, Project administration, Investigation, Funding acquisition, Conceptualization. **Chongzhou Fang:** Writing – original draft, Visualization, Methodology, Investigation, Formal analysis, Conceptualization. **Yulong Qi:** Writing – original draft, Methodology, Investigation, Formal analysis, Conceptualization. **Chong Wang:** Writing – review & editing, Writing – original draft, Supervision, Project administration, Methodology, Investigation, Funding acquisition, Formal analysis, Conceptualization. **Min Wang:** Writing – review & editing, Writing – original draft, Project administration, Conceptualization.

## Ethics approval and consent to participate

All animal procedures were approved by the ethics committee of Peking University Shenzhen hospital ((Approval No 00.2024-333). and conducted in compliance with national guidelines for laboratory animal welfare. The study adhered to the 3R principles (Replacement, Reduction, Refinement). Animals were housed in standard SPF conditions with veterinary supervision. Surgical procedures were performed under anesthesia with postoperative analgesia, and humane endpoints were strictly followed. All personnel received proper training in animal handling and experimental techniques.

## Declaration of competing interest

The authors declare that they have no known competing financial interests or personal relationships that could have appeared to influence the work reported in this paper.

## Data Availability

Data Sharing Not Applicable: The authors do not intend to share the data at this time as these data will be used in several forthcoming studies on Achilles tendon pathology. After the completion and publication of these studies, the authors may consider data sharing upon request. Interested parties should contact the corresponding author via email for inquiries.
